# Characteristics of patients with epithelioid hemangioendothelioma (EHE): a retrospective analysis of the Charité- Universitätsmedizin Berlin

**DOI:** 10.1007/s00432-026-06549-y

**Published:** 2026-07-11

**Authors:** Jana K. Striefler, F. Brandes, A. Strönisch, M. Schmiester, A. Dörr, L. E. Heil Olaizola, J. Benckert, J. Ihlow, A. Jarosch, M. Stiller, M. von Laffert, S. Roohani, D. Kaul, R. Öllinger, S. Wittenberg, D. Rau, S. Märdian, F. Tacke, L. Bullinger, A. Flörcken

**Affiliations:** 1 Department of Internal Medicine II, Oncology/Hematology/BMT/Pneumology, University Medical Center Hamburg-Eppendorf, Hamburg, Germany; 2 Inselspital, Universitätsspital Bern, Universitätsklinik für Medizinische Onkologie, Bern, Schweiz; 3 Department of Hematology, Oncology, and Tumor Immunology, Charité-Universitätsmedizin Berlin, Corporate member of Freie Universität Berlin, Berlin Institute of Health, Campus Virchow-Klinikum, Berlin, Germany; 4 Alexianer St. Hedwig Krankenhaus, Berlin, Germany; 5 Department of Hepatology and Gastroenterology, Charité–Universitätsmedizin Berlin, Corporate member of Freie Universität Berlin, Berlin Institute of Health, Humboldt-Universität zu Berlin, Campus Virchow-Klinikum and Campus Charité Mitte, Berlin, Germany; 6 Charité–Universitätsmedizin Berlin, Corporate member of Freie Universität Berlin, Berlin Institute of Health, Institute of Pathology, Humboldt-Universität zu Berlin, Campus Mitte, Berlin, Germany; 7 Berlin Institute of Health at Charité-Universitätsmedizin Berlin, Berlin, Germany; 8 Department für Diagnostik, Institut für Pathologie, Universitätsklinikum Leipzig AöR, Leipzig, Germany; 9 Department of Radiation Oncology, Charité–Universitätsmedizin Berlin, corporate member of Freie Universität Berlin, Humboldt-Universität zu Berlin, Berlin, Germany; 10 Department of Radiation Oncology, Health and Medical University Potsdam, Potsdam, Germany; 11 Department of Surgery, Charité–Universitätsmedizin Berlin, Corporate member of Freie Universität Berlin, Berlin Institute of Health, Humboldt-Universität zu Berlin, Campus Virchow-Klinikum, Berlin, Germany; 12 Charité–Universitätsmedizin Berlin, Corporate member of Freie Universität Berlin, Berlin Institute of Health, Centre for Musculoskeletal Surgery, Humboldt-Universität zu Berlin, Campus Virchow-Klinikum, Berlin, Germany; 13 Klinik für Unfall-, Hand- und Wiederherstellungschirurgie, Chirurgische Klinik und Poliklinik, Universitätsmedizin Rostock, Rostock, Germany; 14 Partner site Berlin, and German Cancer Research Center (DKFZ), German Cancer Consortium (DKTK), Heidelberg, Germany; 15 National Center for Tumor Diseases (NCT), Partner Site, Berlin, Germany

**Keywords:** Epithelioid hemangioendothelioma, Systemic therapy, Prognostic markers

## Abstract

**Background:**

Epithelioid hemangioendothelioma (EHE) represents an extremely uncommon vascular sarcoma subentity. The clinical characteristics vary from low grade localized to high grade systemic disease and treatment ranges from active surveillance versus intensive therapeutic strategies. Due to its rarity, a standardized clinical management including an appropriate therapeutic approach has not yet been defined.

**Methods:**

Patients with EHE presenting at the Charité-Universitätsmedizin Berlin between 1997 and 2022 were included in the analysis. A retrospective database was established comprising demographic specifications, treatment, pathological features (including TFE3 expression level) and prognosis. Our single-centre data was then compared to previously published cohorts.

**Results:**

The cohort included *n* = 41 patients, with *n* = 16 men (39%) and *n* = 25 women (61%) with a median age of 53 years (range 20–88). The patients had limited comorbidities (median Charlson Comorbidity Index, CCI: 3) and a good performance status (median ECOG: 1). Median overall survival was not reached, with only *n* = 3 patients (7%) showing an aggressive course of disease (progression-free survival (PFS) ≤ 28 days). Isolated liver involvement was the predominant clinical presentation, observed in *n* = 26 patients (63%). At initial diagnosis, *n* = 17 (41%) patients showed systemic symptoms (e.g. anemia, weight loss) and *n* = 12 (29%) already had metastatic disease. Surgery was performed for therapeutic and/or diagnostic purposes in *n* = 20 (49%) patients, whereas only *n* = 5 (12%) received chemotherapy. In *n* = 2 of the latter, disease stabilization was achieved. Liver transplantation was performed in *n* = 5 (12%) patients and was associated with persisting disease-free survival. Molecular genetic data was retrieved in *n* = 21 (51%) patients regarding WWTR1-CAMTA1 fusion, and in *n* = 18 (44%) patients regarding TFE3 positivity. In 67% of analyzed samples, CAMTA1 fusion was found. In contrast, TFE3 expression was observed in 22% only. Both CAMTA1 fusion and TFE3 positivity were observed in *n* = 3 patients (18%).

**Conclusions:**

This analysis of EHE patients treated at Charité-Universitätsmedizin Berlin mirrors published cohorts regarding clinical behavior and OS. The observed clinical heterogeneity underlines the necessity for personalized therapeutic strategies in this rare disease. Molecular characterization is increasingly recognized as a component of contemporary clinical care, as it may identify molecular subclasses associated with aggressive disease and guide management strategies in the future. Moreover, standardized molecular profiling also enables evaluation of patient eligibility for clinical trials.

## Introduction

Epithelioid hemangioendothelioma (EHE) represents an extremely rare sarcoma subentity of endothelial or endothelial stem cell origin. It occurs with a prevalence of < 1:1 000 000 and most often in the fourth to fifth decade of life (Stacchiotti et al. 2021). The median age at initial diagnosis is 36 years, with slightly more females affected (Stacchiotti et al. 2021, Rosenberg and Agulnik 2018). EHE was first described by Weiss and Enzinger as a malignant disease with overlapping features from benign hemangioma and malignant angiosarcoma (Weiss and Enzinger 2018).

On the genetic level, the majority of EHE is characterized by either a t(1;3)(p36.3;q25) translocation resulting in WWTR1-CAMTA1 fusion gene (up to 90%) or a t(X;11)(p11; q22) translocation leading to a fusion of YAP1-TFE3 genes (10%) (Errani et al. 2011, Tanas et al. 2016, Antonescu et al. 2013). The chromosomal rearrangement and the expression of WWTR1-CAMTA1 fusion protein, respectively, disrupts transcriptional regulation resulting in the dysregulation of the Hippo pathway and the subsequent activation of the mitogen-activated protein kinase (MAPK) pathway (Tanas et al. 2016).

Although there are rare cases of WWTR1 (synonym TAZ) translocations involving other genes, CAMTA1 immunohistochemistry (IHC) represents the most reliable surrogate to detect translocation. In contrast, TFE3 IHC is less specific and thus should be confirmed by molecular testing for diagnostic purposes (Errani et al. 2011, Antonescu et al. 2013). To date, none of these molecular genetic aberrations are reliably amenable to pharmacological intervention .

Risk factors for EHE have not been established, but are frequently discussed, including hormonal contraceptives, hepatic injury or inflammation, as well as longtime exposure to asbestos, vinyl chloride, or thorotrast (Treska et al. 2017).

EHE are mostly located in the liver, the lung, or both the lung and the bone. As EHE can arise from vascular structures in every organ, multiple rare localizations have also been described (Rosenberg and Agulnik 2018, Lau et al. 2011). The clinical characteristics vary from indolent to aggressive disease demanding active surveillance versus intensive therapeutic strategies. The median overall survival (OS) from initial diagnosis has been estimated at 4.6 years with a wide range of 6 months to 24 years (Sardaro et al. 2014). Multiple attempts have been made to establish prognostic markers to enable early differentiation between indolent and aggressive disease. Limited disease including multifocal liver disease as well as bone lesions *n* < 2 was associated with a significantly higher 5-year survival rate. Extensive disease, e.g. existence of ascites or pleural effusions either at first diagnosis or throughout the disease course was associated with limited survival rates at 5 years (Lau et al. 2011). Additional parameters such as age > 55 years, male sex, pulmonary metastases, multiple organ involvement, broad infiltration of adjacent organs and systemic symptoms (e.g. anemia or weight loss) have been linked to an inferior outcome (Lau et al. 2011, Sardaro et al. 2014). In contrast, the YAP1-TFE3-fusion has been associated with an indolent clinical course (Antonescu et al. 2013).

Due to the rarity of the disease, a standardized diagnostic and therapeutic approach has not yet been defined. In localized states, complete resection is the standard of care (Rosenberg and Agulnik 2018). There is also some retrospective data on the use of radiation therapy and other locoregional treatments, e.g. percutaneous ablation, transarterial chemoembolization, radioembolization, and ischemic limb perfusion in selected cases (Kamarajah et al. 2018, Høyer et al. 2012, Kou et al. 2020, Cardinal et al. 1960).

In multifocal liver disease without extrapulmonary distant metastases, evaluation of liver transplantation is recommended as it provides a curative option with long term survival (Mehrabi et al. 2006). As spontaneous remissions have been described, watchful waiting may be considered in asymptomatic disease or for patients not qualifying for surgery due to comorbidities or technical challenges (Stacchiotti et al. 2021, Kitaichi et al. 1998). In advanced or metastatic EHE, no standard therapy has been established. Due to its endothelial origin, vascular endothelial growth factor receptor (VEGFR)-directed therapies such as bevacizumab or pazopanib are being utilized. Conventional chemotherapy shows only limited activity and thus is reserved for particularly aggressive cases comparable with high grade soft tissue sarcomas (STS).

In retrospective analyses, antitumor activity was documented with interferon, thalidomide, multi-tyrosine kinase inhibitors and mechanistic target of rapamycin (mTOR) inhibitors, the latter showing the highest clinical impact regarding overall survival (OS) as well as progression-free survival (PFS) (Stacchiotti et al. 2021). In accordance with preclinical data suggesting activation of the MAPK signaling pathway through CAMTA1 fusion, the MEK inhibitor trametinib demonstrated objective tumor response in 9.5% of patients and a significant reduction of symptoms in preliminary results from a phase II trial (Schuetze et al. 2021). Furthermore, there is an ongoing phase II clinical trial with the mitotic inhibitor Eribulin (NCT03331250), as well as a phase II clinical trial testing an oral *transcriptional enhancer associate domain* (TEAD) inhibitor targeting the hippo signaling pathway (NCT05228015).

The advent of TEAD inhibitors is a major recent development, which aim to block YAP/TAZ-TEAD–dependent transcription and demonstrate promise in preclinical and early clinical studies (Pobbati et al. 2023). These agents represent a rational targeted approach for tumors driven by Hippo pathway dysregulation, as seen in EHE with characteristic gene fusions (Suto 2024).

Parallel to therapeutic progress, international registry initiatives have been established to advance EHE research. Notably, the pan-European EHE Prospective Registry, supported by EURACAN, was launched to systematically collect clinical and molecular data across multiple centers (Frezza et al. 2024). This registry seeks to enhance the understanding of EHE’s natural course, improve prognostic classification and therapeutic response assessment, promote consistency in clinical management, and generate real-world evidence to support future clinical trials.

These advances position the current study within a rapidly evolving landscape that integrates precision therapies and collaborative registry-based research efforts in the management of EHE.

We performed a single-center retrospective analysis to address the following research questions:

- What are the clinical, pathological, and molecular characteristics of patients diagnosed with epithelioid hemangioendothelioma (EHE) and treated at Charité–Universitätsmedizin Berlin between 1997 and 2022?
- How do clinical outcomes, including overall survival and disease progression, in this single-center cohort compare with those reported in previously published international studies?
- Is there an association between specific molecular alterations—particularly WWTR1-CAMTA1 fusion and TFE3 expression—and disease aggressiveness or distinct clinical behavior?

Based on these questions, the study hypothesizes that molecularly defined subgroups of EHE, characterized by WWTR1-CAMTA1 fusion or TFE3 expression, exhibit divergent clinical courses and prognostic outcomes, supporting the need for molecularly guided, personalized therapeutic strategies.

Our findings underline the high unmet need to establish prognostic markers and to improve therapeutic strategies.

## Patients and methods

### Patients

All patients with a diagnosis of epithelioid hemangioendothelioma (EHE) who received care at Charité – Universitätsmedizin Berlin between 1997 and 2022 were considered eligible for inclusion in this retrospective study. Case identification was performed through a systematic search of the institutional pathology database using standardized diagnostic categories, supplemented by extraction of cases from the hospital’s electronic clinical information system based on relevant ICD codes. Each potential case was independently reviewed by two experienced physicians (a board-certified pathologist and a medical oncologist) to confirm the diagnosis according to established histopathological and immunohistochemical criteria (including, where available, evidence of CD31, ERG, FLI-1, CAMTA1, or TFE3 expression) and to exclude duplicates, patients with incorrect diagnoses, or misassigned codes.

Comprehensive demographic, clinical, treatment, and outcome data were collected through a combination of electronic medical records review and manual inspection of archived clinical files, ensuring that only cases with sufficiently complete datasets were included in the final analysis.

All patients diagnosed and/or treated for epithelioid hemangioendothelioma (EHE) at our Sarcoma Center during the defined study period and with complete records were included. Patients with incomplete records were excluded from analysis. Records were defined as incomplete if one or more of the following core inclusion variables were missing: patient name, sex, date of birth, diagnosis, date of diagnosis, or date of first presentation at the Sarcoma Center. Only cases with complete information for all of these mandatory variables were considered eligible for inclusion.

To ensure transparency, the extent of missing data for each key variable is summarized in the respective tables. A dedicated column labeled “not available” indicates the number of cases with missing values for individual items, enabling assessment of data completeness across the dataset.

Missing data were handled by complete-case analysis. Only subjects with available data for the relevant variables were included; missing values were not imputed. Imaging data were obtained as part of routine clinical diagnostics in a real‑world setting. Due to the retrospective nature of the study, imaging schedules were not fully standardized, and examinations were not consistently performed with identical imaging equipment or by the same radiologists. In several cases, imaging studies originated from external institutions and were incorporated into the clinical record. All cases were, however, systematically reviewed within the institutional sarcoma tumor board, where imaging findings were presented and evaluated by board‑certified radiologists with expertise in sarcoma imaging.

Localized disease was defined as cancer confined to the organ or tissue of origin without evidence of spread beyond the primary site. Metastasized disease was defined as cancer that had spread to distant organs or tissues, confirmed by imaging or pathology. Disease progression was defined based on clinical and/or radiological evidence as documented in the patients’ medical records, with information obtained from radiology reports, clinical notes, and follow-up documentation describing disease course or parameters indicative of progression. Data collection and analysis was in line with local ethical guidelines and according to the local ethical vote (EA2/240/20). In addition to clinical parameters, comprehensive data regarding the histology, including immunohistochemistry and molecular pathology, was collected in cooperation with the Institute of Pathology of Charité – Universitätsmedizin Berlin. If not available from routine diagnostics, IHC for TFE3 and FISH or RNA sequencing for WWTR1-CAMTA1 fusions was complemented within a collaboration project with the Institute of Pathology of Leipzig, Germany.

**Fig. 1:**
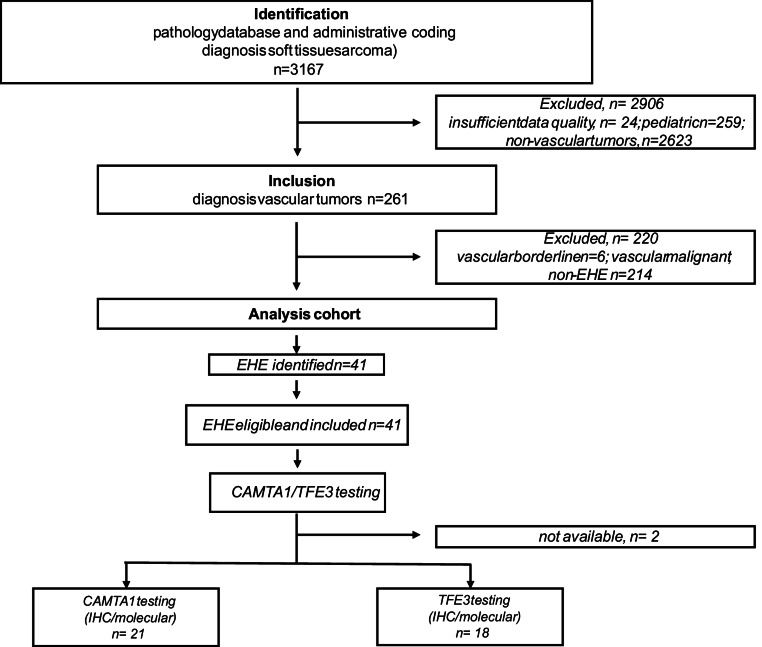
Study flow diagram. Exact case numbers for some intermediate extraction steps could not be fully retrieved from the historical database; this limitation is acknowledged

### Immunohistochemistry

For TFE3 immunohistochemistry, sections of paraffin-embedded EHE samples were stained using automated Ventana BenchMark XT immunostainer (Ventana Medical Systems, Inc., Tucson, AZ, USA). Briefly, the tissue sections were deparaffinized, rehydrated and subjected to heat-induced epitope retrieval and endogenous peroxidase blocking using H2O2 (program: CC1 mild). Subsequently, EHE-slides were incubated for 32 min with the TFE3 antibody (Cell Marque, clone MRQ-37, catalogue number 760–4622, ready to use). This was followed by chromogen 3,3′-diaminobenzidine tetrahydrochloride (UV-DAB) application for 8 min and counterstaining with haematoxylin. Whole slides were examined by two pathologists using an Olympus BX46 microscope. Strong, moderate and mild nuclear staining results were defined as TFE3-positive. TFE3 positivity was defined as the presence of TFE3 nuclear expression in at least 10% of tumor cells, with at least weak staining intensity, as previously described (Sharain et al. 2019). Lack of nuclear staining was considered TFE3-negative.

### Molecular analyses by FISH und RNA sequencing

Interphase Fluorescence in-situ Hybridization (FISH) for WWTR1 was conducted using a dual color break apart probe (Zytovision, catalogue number Z-2212-50) targeting split signals at the chromosome region 3q24-3q25.1 that harbors the WWTR1 (WW domain containing transcription regulator 1, a.k.a. TAZ) gene. Sequences mapping in 3q24-q25.1 (chr3:148,533,200-149,234,601) proximal to the WWTR1 break point were targeted with ZyGreen chromogen. Sequences mapping in 3q25** (chr3:149,430,325-149,933,565) distal to the WWTR1 breakpoint region were targeted using ZyOrange chromogen. Split signals were counted in 100 cells. A cut-off 10% (minimum 10 split signals) was considered positive. Additionally, if fusions could not be detected by IHC or ISH, archival tumor tissue was dissected and total RNA extracted. Targeted fusion analysis was realized with Next Generation Sequencing Technology (MiSeq, Illumina) using Customized QIAseq Targeted RNAscan Panels (Qiagen).

Initially, cDNA synthesis and subsequently amplification of known breakpoint regions of 138 fusion genes was performed. Please refer “Anlage 3_Genliste RNA Scan Sarcoma” on the webpage of Institute of Pathology of University Hospital Leipzig (https://www.uniklinikum-leipzig.de/einrichtungen/pathologie/Freigegebene%20Dokumente/1.3%20Anhang%203%20Genliste%20RNA%20Scan%20Sarcoma.pdf) for more detailed information on extent of analyzed gene regions. Bioinformatic analysis was executed with SEAMLESS NGS software (EcSeq). Selective fusion calling was conducted with minimal fusion support of three Split-Reads. Tissue specific gene expression of the respective fusion partner was undertaken by literature research and data bank analysis (e.g. GTEx-Portal) and then integrated into the validation.

### Statistical analysis

Overall survival (OS) and progression-free survival (PFS) were calculated from the date of diagnosis to death or to the first event of progression, respectively. Although some patients were initially diagnosed externally and referred to our center at variable time intervals, this approach was chosen to ensure comparability across the entire cohort.

For PFS, an event was defined as local relapse, distant relapse, or death. OS and PFS curves were calculated using the Kaplan–Meier estimator with log-rank tests and proportional hazards regression for univariable survival analyses. In the univariable Cox regression analyses, the proportionality assumption was systematically tested for each variable using Schoenfeld residuals. For progression-free survival, the proportionality assumption was violated for the variables *sex* and *surgical therapy*, indicating that their effects diminished over time. Accordingly, a time-dependent Cox model was applied. All statistical analyses were performed as complete-case analyses, including only patients with available data for the respective variables. Given the small cohort size, results must be interpreted with caution; therefore, only hazard ratios at one month are reported for time-dependent variables. Chi-square tests were used to examine associations between categorical variables. In general, P values were calculated two-sided and considered significant when < 0.05. Data analysis was performed using IBM SPSS Statistics (version 29) and R software (version 4.3.2).

## Results

### Patient and tumor characteristics

Data of *n* = 41 patients were available for analysis. Median follow-up was 92 months (0.5-329.6). The majority of patients were female (*n* = 25, 61%). Median age at diagnosis was 53 years (range 20–88 years). Most patients, aside from their underlying malignancy, presented with an overall favorable clinical condition (median CCI: 3) and a maintained performance status (median ECOG: 1). In patients with known liver lesions (*n* = 27, 66%), we also documented bilirubin and creatinine as surrogate parameter for liver and kidney function. Most commonly, liver function was preserved.

For details on patient characteristics, please refer to Table 1.

**Table 1:** Baseline patient characteristics

Characteristic	
Age Median, years (range)	53 (20–88)
Sex n, (%)	
Female	25 (61)
Male	16 (39)
CCI median (range)	3 (0–11)
ECOG median (range)	1 (0–3)
Creatinine (mg/dL)* (range)	0.84 (0.62–5.08)
Bilirubin (mg/dL)* (range)	0.47 (0.16–12.38)
Systemic symptoms (e.g. anemia, weight loss) n, (%)	n = 41
Yes	17 (41)
No	17 (41)
Data not available	7 (17)
LDH elevation (>250 U/l) n, (%)	n = 41
Yes	8 (20)
No	21 (51)
Data not available	12 (29)
Thrombocytes/nl median (range)	244 (51–617)
Esophageal varices n, (%)	n = 41
Yes	4 (10
No	21 (51)
Data not available	16 (39)

CCI, Charlson Comorbidity Index; ECOG. Eastern Cooperative Oncology Group Performance Status

*only calculated for patients with hepatic involvement

In our cohort, most patients presented with hepatic manifestation (*n* = 26, 63%) or localized disease (*n* = 26, 63%). In ten patients (24%), pleural and/or peritoneal effusions were observed; these developed during the course of the disease and were not present at initial diagnosis. Detailed tumor characteristics are listed in Table 2.

**Table 2:** Baseline tumor characteristics

Characteristic	
Localization n, (%)	n = 41
Liver only	26 (63)
Lung only	3 (7)
Skin only	1 (2)
Other (soft tissue, bone, vascular)	6 (15)
Data not available	3 (7)
Stage n, (%)	n = 41
Localized	26 (63)
Metastasized	11 (27)
Data not available	4 (10)
Metastases lung and liver only n, (%)	n = 41
Yes	10 (24)
No	28 (68)
Data not available	3 (7)
Metachronous metastases n, (%)	n = 41
Yes	12 (29)
No	26 (63)
Data not available	3 (7)
Angioinvasion n, (%)	n = 41
Yes	8 (20)
No	9 (22)
Data not available	24 (59)
Pleural involvement n, (%)	n = 41
Yes	5 (12)
No	34 (83)
Data not available	2 (5)
Ascites	n = 41
Yes	5 (12)
No	31 (76)
Data not available	5 (12)
Pleural and/or peritoneal effusion	n = 41
Yes	10 (24)
No	26 (63)
Data not available	5 (12)
Hemoptysis	n = 41
Yes	0 (0)
No	39 (95)
Data not available	2 (0)

A comprehensive molecular analysis for CAMTA1 fusion was performed in *n* = 21 patients (51%). Of those, the majority (*n* = 14, 67%) had a CAMTA1 fusion. TFE3 positivity was analyzed by immunohistochemistry in *n* = 18 patients (44%). In only *n* = 4 patients (22%) TFE3 expression was observed. Both molecular genetic markers were positive in *n* = 3 patients (18%). Table 3 lists all molecular characteristics of the cohort. Four cases were negative for both CAMTA1 and TFE3. In half of them (*n* = 2), RNA quality was limited, resulting in less representative results.

**Table 3:** Molecular characteristics

CAMTA1 fusion n, = (%)	n = 21
Yes	14 (67)
No	2 (9)
Not evaluable	5 (24)
TFE3 positivity n, = (%)	n = 18
Yes	4 (22)
No	14 (78)
TFE3 positivity and CAMTA1 fusion n, = (%)	n = 17
Yes	3 (18)
No	14 (82)

There was no association between genetic aberrations (CAMTA1 fusion, TFE3 positivity, both) and the following parameters: systemic symptoms, stage, sex, age, CCI, and effusion (data not shown).

Slightly fewer than half of the patients received surgery (*n* = 20, 49%) with diagnostic and/or therapeutic intent, with two procedures performed solely for diagnostic purposes and all remaining interventions consisting of extensive tumor resections.

*N* = 5 patients (12%) underwent liver transplantation. The mean waiting time for liver transplantation from listing to transplantation was 540 days, ranging from 57 to 1,151 days, with a median of 484 days. The patient with the notably short waiting time of 57 days underwent living donor liver transplantation. During the study period, allocation criteria underwent several modifications. In 2006, the Model for End-Stage Liver Disease (MELD) system was introduced. For patients transplanted prior to its implementation (*n* = 3) organs were allocated based on historical urgency criteria, which also incorporated waiting time as a relevant factor. Following the introduction of the MELD system, exception-based allocation pathways were established for certain oncological indications, such as hepatocellular carcinoma (HCC), referred to as standard exceptions (SE). For epithelioid hemangioendothelioma (EHE), a standard exception was introduced in 2010. The patient transplanted after the introduction of the MELD system but prior to the establishment of the SE for EHE received an individually approved exception (non-standard exception, NSE). With the introduction of the standard exception for EHE, strict eligibility criteria were defined. Patients were required to be listed for at least one year and to demonstrate absence of extrahepatic disease at the time of exception application, thereby confirming liver-limited disease over a minimum period of one year. After approval of the SE, continuation of the SE required reassessment every three months, including imaging to exclude extrahepatic manifestations. Accordingly, patients underwent cross-sectional imaging (CT) at three-month intervals. A similar surveillance strategy had already been applied prior to the introduction of the SE. In the NSE application following MELD implementation, the disease was likewise described as confined to the liver with slow progression over an extended period. For the three earliest transplant recipients, extrahepatic disease was excluded both at initial diagnosis and immediately prior to transplantation. Patient selection was consistently based on favorable tumor biology characterized by indolent progression. Surveillance was performed largely in accordance with criteria later formalized in the SE framework.

Similarly, even before the introduction of the standard exception, patient selection and listing practices followed comparable principles.

Radiation therapy was performed in 2 patients (5%), ranging from external beam radiotherapy of the primary tumor region with a cumulative dose of 66 Gy and interstitial liver brachytherapy in one patient to combined radiofrequency ablation and interstitial brachytherapy for hepatic metastases in another, while a further patient received radiofrequency ablation of hepatic metastasis.

Only 6 patients (15%) were treated with systemic therapy, which consisted of conventional chemotherapy with paclitaxel (1–5 cycles) or trofosfamide (maintenance therapy for approximately 28 months) in 5 patients (12%) and immunomodulatory therapy with interferon-alpha in 1 patient (2%); antiangiogenic agents and tyrosine kinase inhibitors were not administered. For details on the therapeutic strategy, see Table 4.

**Table 4:** Therapeutic strategy

Type/Modality	
Surgery n, = (%)	n = 41
Yes	20 (49)
No	18 (44)
Data not available	3 (7)
Liver transplantation n, = (%)	n = 41
Yes	5 (12)
No	33 (80)
Data not available	3 (7)
Radiation therapy n, = (%)	n = 41
Yes	2 (5)
No	32 (78)
Data not available	6 (15)
Radiofrequency ablation	n = 41
Yes	1 (2)
No	31 (76)
Data not available	6 (15)
Systemic therapy any n, = (%)	n = 41
Yes	6 (15)
No	28 (68)
Data not available	7 (17)
Chemotherapy n, (%)	n = 41
Yes	5 (12)
No	30 (73)
Data not available	6 (15)
Antiangiogenic therapy n, = (%)	n = 41
Yes	0 (0)
No	35 (85)
Data not available	6 (15)
Immunomodulatory agents n, (%)	n = 41
Yes	1 (2)
No	35 (85)
Data not available	5 (12)
Tyrosine kinase inhibition n, = (%)	n = 41
Yes	0 (0)
No	36 (88)
Data not available	5 (12)

### Outcome

**Fig. 2:**
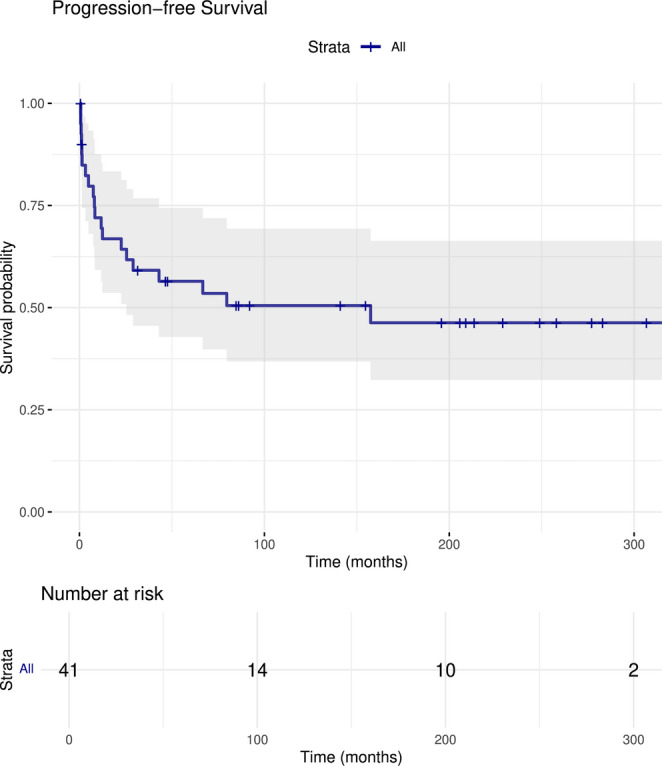
Kaplan–Meier overall study population (PFS)

At the time of data cut-off, the median follow-up was 92 months (range: 0.5–329.6 months). Twelve patients (29%) had died, all of whom had progressive disease (PD) (median overall survival [OS]: not reached, 95% CI: not available). In total, 20 patients (48%) experienced progressive or refractory disease (median progression-free survival [PFS]: 157 months, 95% CI: not available; see Figs. 1 and 2).

Three patients within the cohort experienced notably rapid clinical deterioration. Patient 1 (32 years) presented after a self‑imposed 9‑month diagnostic delay with severe weakness, weight loss, and advanced hepatic dysfunction (hepatorenal syndrome, ascites), requiring dose‑reduced systemic therapy. Patient 2 (63 years) had been diagnosed 12 years before treatment initiation; at recurrence, he developed progressive dyspnea, pain, and weight loss over 3 months, with systemic therapy commenced 8 months after re‑evaluation. Patient 3 (50 years) presented in 2006 with peritoneal effusion, hepatorenal failure, and a markedly impaired performance status; liver transplantation was considered but contraindicated due to metastatic and decompensated disease, and best supportive care was provided. All three patients demonstrated fulminant progression with rapid functional decline.

**Fig. 3:**
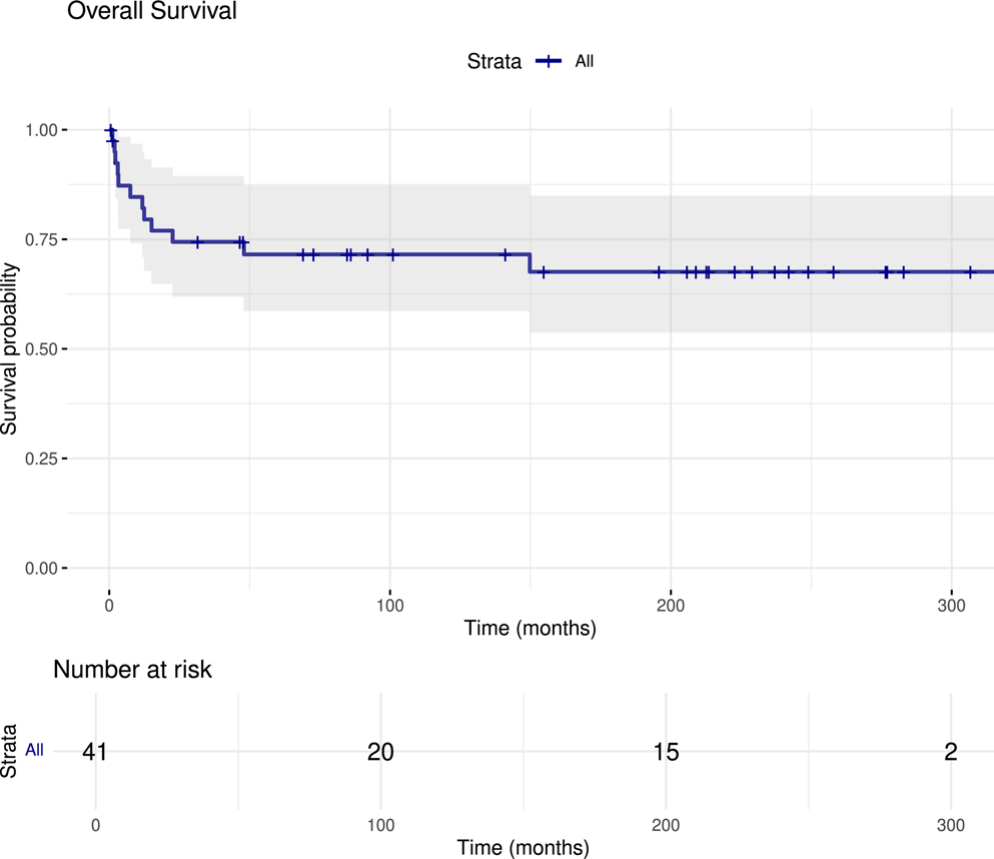
Kaplan–Meier overall study population (OS)

### Prognostic parameters

**Fig. 4:**
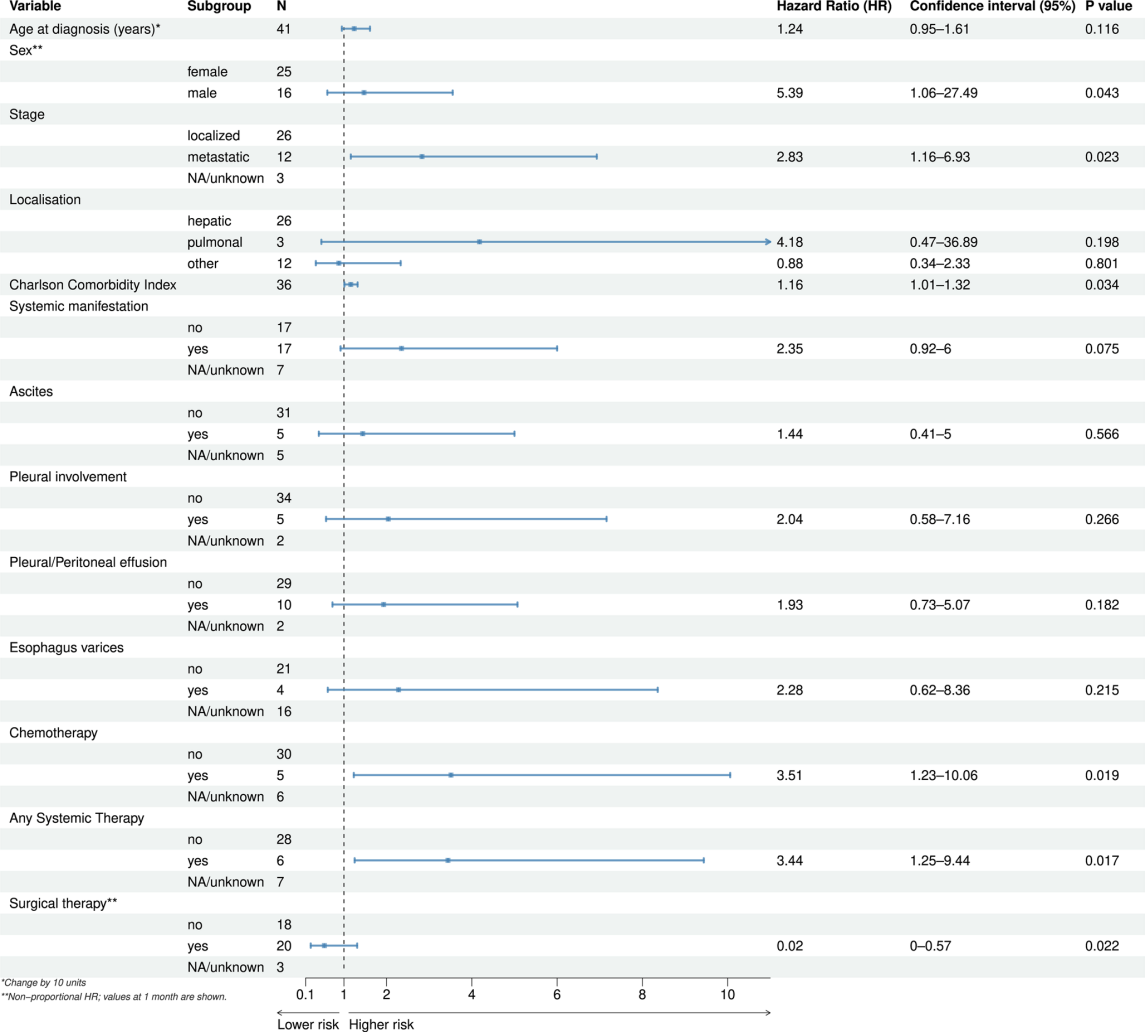
Forest plot of progression free survival (PFS). Cox regression–derived hazard ratios with 95% confidence intervals

In our cohort, regarding progression-free survival, significant differences were found for sex (*p*=.043), stage (*p*=.023), CCI (*p*=.034), chemotherapy (*p*=.019), any systemic therapy (*p*=.017) and surgical therapy (*p*=.022). Please refer to Fig. 4.

**Fig. 5:**
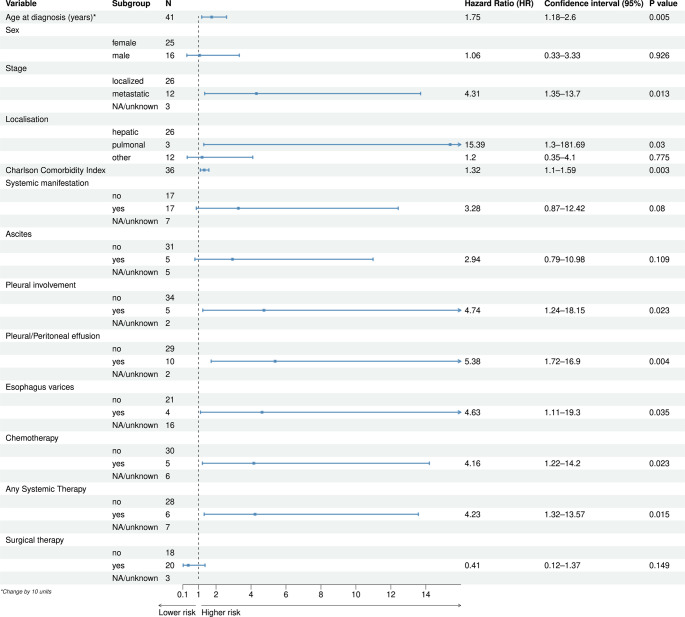
Forest plot of overall survival (OS). Cox regression–derived hazard ratios with 95% confidence intervals

Overall survival was significantly influenced by age (*p*=.005), stage (*p*=.013), localisation (*p*=.03), CCI (*p*=.003), pleural involvement (*p*=.023), serous effusion (*p*=.004), presence of esophageal varices (*p*=.035), chemotherapy (*p*=.023), and any systemic therapy (*p*=.015). Please refer to Fig. 5.

Of the *n* = 5 patients who underwent liver transplantation, none experienced recurrence, with a median follow-up of 19 years (range, 11–22 years). Postoperative complications related to transplantation were observed: four out of five patients experienced a single episode of mild acute cellular rejection within the first year after transplantation. Two patients required surgical revision (hematoma evacuation on postoperative day 2 and revision of a kinking stenosis of the hepatic artery four months after transplantation). One patient underwent angiographic partial occlusion of the splenic artery due to splenic steal syndrome and later required herniotomy for an incisional hernia.

Consistent with previously published data, we found prognostic differences depending on sex, CCI ≥ 3, age ≥ 65 years, but not for TFE3 positivity and CAMTA1 mutational status.

## Discussion

Our EHE cohort comprised 41 patients with a median age of 53 years and a well-preserved performance status (median ECOG = 1). Hepatic manifestation and localized disease were the most common presentations. Even in patients with hepatic manifestations, liver and kidney function were rarely impaired. Elevated bilirubin and/or creatinine levels were uncommon, with median bilirubin at 0.47 mg/dL and median creatinine at 0.84 mg/dL.

Age, stage, localisation, CCI, pleural involvement, and the presence of esophageal varices, effusion, chemotherapy as well as any systemic therapy were associated with reduced overall survival, whereas no clear associations were observed for TFE3 and CAMTA1 mutational status; however, these results should be interpreted with caution given the limited number of events and the wide confidence intervals. Previously described risk factors were assessed based on the available information; however, tumor size and mitotic count could not always be determined due to the predominance of biopsy samples (Deyrup et al. 2008). In contrast, some of the known clinical predictors with prognostic impact including symptomatic disease (e.g. effusions), as well as stage and localisation, were confirmed in our analysis (Stacchiotti et al. 2023, Liu and He 2022, Tomassen et al. 2023).

The median age in our patient population corresponded to other cohorts (53 vs. 45 years) (Frezza et al. 2021).

With regard to surgical therapy, it should be noted that our clinic is a high-volume center with outstanding surgical expertise, including a large transplantation unit. This undoubtedly impacts both patient referral patterns and, potentially, treatment decisions, resulting in a selection bias.

Overall, all transplanted patients exhibited a favorable clinical course, with a low complication rate and excellent long-term survival compared to the overall transplant cohort at our center. The overall 5-year survival rate following liver transplantation for all indications at our center ranges from 64.3% to 77.1%, depending on the era of transplantation (Ritschl et al. 1929).

In our analysis, receiving systemic therapy was associated with worse overall survival. However, this apparent association is most likely driven by confounding by indication, as drug therapy was predominantly administered to patients with symptomatic disease and high tumor burden. Moreover, because the exact timing of treatment initiation was not consistently available, our analysis is at risk of immortal time bias. Consequently, these results must not be interpreted as demonstrating a harmful causal effect of systemic therapy on overall survival, but rather as a reflection of underlying differences in disease severity and treatment allocation. This patient group inherently has a much poorer prognosis, regardless of the treatment administered.

A small subset of three patients displayed fulminant clinical progression, characterized by rapid functional decline and limited therapeutic tolerance. Their presentations were marked by delayed diagnosis, extensive hepatic involvement, and poor performance status at baseline—factors that likely contributed to their unfavorable course. In Patient 1, a prolonged delay before initial presentation may have allowed clinically silent progression to critical organ dysfunction. Patients 2 and 3 similarly exhibited advanced disease at the time of treatment consideration, underscoring the narrow therapeutic window in rapidly progressive cases. These observations highlight a distinct clinical phenotype of aggressive disease biology or host vulnerability, suggesting that early recognition and timely intervention may be crucial to improving outcomes in this subgroup.

Despite current recommendations for mTOR blockade (e.g., sirolimus) and VEGFR inhibition (e.g., pazopanib) in symptomatic disease, and although a large proportion of patients in our cohort presented with disease-related symptoms, both targeted agents were rarely administered (Stacchiotti et al. 2021). This likely reflects the extended study period (1997–2022), during which diagnostic and therapeutic standards for EHE evolved substantially. In the earlier years, molecular diagnostic techniques were not yet routinely available, and the interdisciplinary Sarcoma Center at Charité–Universitätsmedizin Berlin was still in development. The publication of the EHE consensus paper and the broader adoption of standardized molecular testing only occurred in recent years, improving diagnostic precision and therapeutic awareness (Stacchiotti et al. 221). Moreover, mTOR inhibitors and VEGFR inhibitors were introduced gradually and initially approved for other tumor entities, with pazopanib, for example, receiving FDA approval for soft tissue sarcomas only around 2012. These temporal changes in diagnostic and therapeutic opportunities may have influenced treatment allocation and outcomes, and likely contribute to the heterogeneity observed in the present cohort.

Unlike Stacchiotti et al., we observed no difference in clinical behavior when WWTR1-CAMTA fusion was present (e.g. stage, systemic manifestations) (Stacchiotti et al. 2023). It should be noted, however, that this finding must be interpreted with caution. In our cohort—which includes several cases diagnosed externally and/or from earlier years—molecular testing was not available for all patients, and the overall number of genetically characterized cases was limited. Therefore, the absence of a prognostic association for CAMTA1 and TFE3 status in our analysis should be considered inconclusive. Consistent with this, we observed a comparatively lower proportion of patients with WWTR1‑CAMTA1 fusion (67% vs. 90%) and a higher rate of TFE3 positivity (22% vs. 10%) compared to Stacchiotti et al.

Several factors may also contribute to the lower observed frequencies of CAMTA1 fusions in our cohort. Tumor heterogeneity and intrinsic biological variability may play a role, and limited residual tissue precluded retrospective full-panel fusion testing in all cases. Differences in assay sensitivity ─whether using targeted RNA panels, FISH, or other molecular methods ─may also limit detection of uncommon or variant fusion partners, potentially underestimating true CAMTA1 positivity. Notably, four cases were negative for both CAMTA1 fusion and TFE3 IHC, reflecting either exceptionally rare biological events or, more likely, technical limitations in both molecular and immunohistochemical assays. Our fusion-gene assay included 138 potential fusion partners, including WWTR1, CAMTA1, and YAP1, enabling detection of both canonical and rare rearrangements. This broad coverage reduces the likelihood that uncommon CAMTA1 fusions were missed and provides substantially higher sensitivity than CAMTA1 immunohistochemistry. TFE3 status was assessed by IHC because TFE3 fusion testing was not routinely available at the time of case workup. We acknowledge that TFE3 IHC positivity alone is not equivalent to molecular confirmation of a TFE3 fusion and may yield false-positive results. The limitations of TFE3 IHC caused by fixation, decalcification, antibody performance, and interpretive subjectivity are therefore recognized. Particularly for TFE3-IHC, this results in limited specificity and may explain the weak nuclear TFE3 positivity observed in three CAMTA1-fusion–positive cases, though rare TFE3 fusions cannot be fully excluded(Argani et al. 2003, Rao et al. 2013, Tretiakova 2022). To minimize variability, IHC in our laboratory is performed on standardized, validated stainers under tightly controlled conditions, with regular participation in external quality-assessment schemes. Confirmatory molecular testing was not feasible for all cases; however, in those analyzed with fusion panels, YAP1–TFE3 fusions were indirectly covered as YAP1 was included in the assay design. However, future studies should apply comprehensive fusion-gene assessment to all EHE samples, in accordance with current recommendations (Stacchiotti et al. 2023, CDM et al. 2013). Despite the limitations mentioned above, our data suggest that TFE3 positivity and WWTR1-CAMTA fusion, as previously described, are not mutually exclusive (Lee et al. 2016). Nevertheless, EHE showing negativity for both TFE3- and CAMTA1-fusion are extremely rare events and should be interpreted with caution (CDM et al. 2013).

Limitations of this analysis are the small size of the study population and the retrospective character with partially incomplete patient data and biases inherent to retrospective analyses.

For example, some pathological characteristics, tumor size and pain were not documented.

The time between diagnosis and presentation at our institution was highly heterogeneous and not prognostically meaningful due to the evolving therapeutic landscape and changes in referral patterns over the extended study period. Therefore, the date of diagnosis was considered the most appropriate and consistent starting point for survival analyses in this cohort.

In addition, the lack of a standardized imaging schedule represents an inherent limitation of the retrospective, real‑world study design. Although imaging protocols and equipment varied between institutions, the consistent review and discussion of imaging studies by specialized radiologists within a multidisciplinary tumor board ensured a uniform clinical interpretation and minimized diagnostic variability.

All statistical analyses were performed as complete-case analyses, including only patients with available data for the respective variables. This approach may have introduced selection bias and could limit the generalizability of the results. Due to incomplete molecular data, some analyses were only performed recently for our study, using modern protocols and equipment. Therefore, methods used for genetic diagnostics are heterogenous and encompass IHC, FISH, RT-PCR and RNAseq. Comparability of the results can nevertheless be assumed, as TFE3 positivity can be determined using all of the mentioned methods (Habeeb and Rubin 2019). However, the situation is different for fusion of WWTR1 and CAMTA1. This can only be detected using FISH, RT-PCR and RNAseq.

In addition to clinical data, quality of life urgently needs to be examined in patients with EHE, constituting an integral factor for therapeutic decisions given the unpredictable clinical behavior of this entity. To date, there is no established systemic standard for the treatment of EHE. Therefore, the identification of prognostic and predictive biomarkers is all the more important. Standard regimens for soft tissue sarcoma are not effective. Potential therapeutic options include trametinib (NCT03148275) (Schuetze et al. 2021), and immunotherapy with or without sunitinib (IMMUNOSARC, NCT03277924). In addition, dose escalation of sirolimus depending on course of disease is tested (Stacchiotti et al. 2023).

Because of the limited and often ineffective therapeutic options available for this disease, precise patient selection would be of crucial importance. At present, however, there are no known criteria for distinguishing between rapid and aggressive versus slow and mildly symptomatic disease courses. From our perspective, it is therefore of fundamental importance to better understand EHE biology. We will continue to pursue this in follow-up projects.

To further expand the knowledge of EHE and to generate an external control for studies without a control cohort, patient registries may represent a fundamental tool (Stacchiotti et al. 2021). Only recently, an international registry for EHE within the EURACAN network was initiated by Trama et al. (NCT06408441). Our analysis provides an important contribution to a better understanding of this disease due to the relevant size of the cohort and the fact that it describes real-life care.

## Data Availability

All data generated or analyzed during this study are included in this article. Further enquiries can be directed to the corresponding author.

## Abbreviations

95%CI: 95% confidence interval
CAMTA1: Calmodulin binding transcription activator 1
CCI: Charlson comorbidity index
e.g.: Exempli gratia
ECOG: Eastern Cooperative Oncology Group
EHE: Epithelioid hemangioendothelioma
IHC: Immunohistochemistry
MAPK: Mitogen-activated protein kinase
mTOR: Mechanistic target of rapamycin
NR: Not reached
NS: Not significant
OS: Overall survival
PFS: Progression-free survival
RNA: Ribonucleic acid
RNAseq: RNA sequencing
STS: Soft tissue sarcoma
TFE3: Transcription factor E3
VEGFR: Vascular endothelial growth factor receptor
vs: Versus
WWTR1: WW domain-containing transcription regulator 1
YAP1: Yes-associated protein 1
TEAD: Transcriptional enhancer associate domain
